# Weather, weekday, and vacation effects on webcam recorded daily visitor numbers in the alpine winter season

**DOI:** 10.1007/s00484-023-02591-4

**Published:** 2023-12-01

**Authors:** Simon Kloos, Carina Bigalke, Matthias Neumair, Annette Menzel

**Affiliations:** 1 TUM School of Life Sciences, Ecoclimatology, Technical University of Munich, 85354 Freising, Germany; 2 TUM School of Life Sciences, Life Science Systems, Technical University of Munich, 85354 Freising, Germany; 3 Institute for Advanced Study, Technical University of Munich, 85748 Garching, Germany

**Keywords:** Winter tourism, Skiing, Meteorology, Weekend, Vacation, Object detection

## Abstract

Winter tourism is an important economic factor in the European Alps, which could be exposed to severely changing meteorological conditions due to climate change in the future. The extent to which meteorology influences winter tourism figures has so far been analyzed mainly based on monthly or seasonal data and in relation to skier numbers. Therefore, we record for the first time daily visitor numbers at five Bavarian winter tourism destinations based on 1518 webcam images using object detection and link them to meteorological and time-related variables. Our results show that parameters such as temperature, cloud cover or sunshine duration, precipitation, snow depth, wind speed, and relative humidity play a role especially at locations that include other forms of winter tourism in addition to skiing. In the ski resorts studied, on the other hand, skiing is mostly independent of current weather conditions, which can be attributed mainly to artificial snowmaking. Moreover, at the webcam sites studied, weekends and vacation periods had an equal or even stronger influence on daily visitor numbers than the current weather conditions. The extent to which weather impacts the (future) visitor numbers of a winter tourism destination must therefore be investigated individually and with the inclusion of non-meteorological variables influencing human behavior.

**Supplementary information:**

The online version contains supplementary material available at 10.1007/s00484-023-02591-4.

## Introduction

Tourism has a high economic and social impact on the European Alpine region (Bätzing 2015; Bausch & Gartner 2020; Pröbstl-Haider et al. 2019). However, in future climate change scenarios, negative impacts on skiing and winter tourism, such as reduced reliability of slopes that depend on natural snow, less possibilities to produce artificial snow, shortened and more variable skiing seasons, and a decrease in the number of operating ski resorts, prevail (Gilaberte-Búrdalo et al. 2014, Steiger et al. 2019, Steiger et al. 2022 and especially for Bavaria: Mayer & Steiger 2013). With a global warming of only 2°, the weather-related risk of losses in winter overnight stays associated with ski tourism in Europe is projected to be as high as 10.1 million overnight stays per winter season (Damm et al. 2017).

The relationship between meteorology and winter tourism and especially ski tourism numbers has been intensively studied. In multiple studies, the influence of snow conditions and/or weather on ski tourism has been confirmed (Demiroglu et al. 2015; Mayer and Abegg 2022; Shih et al. 2009). However, it is necessary to distinguish between the magnitude and nature of the relationship. While Koenig and Abegg (1997), Steiger (2011) and Pickering (2011) found in some cases considerable effects of winters with little snow on ski tourism in Switzerland, Austria, and in Australia, respectively, the effects of different weather variables were rather small in other studies (e.g., Falk 2013, 2015). It is also clear that the influence of weather and in particular snow depth on ski tourism is directly related to the altitude of the ski area studied: While the dependency between weather and tourist numbers is higher especially in low altitude ski resorts with higher average temperatures and a lower snowmaking potential, higher altitude ski resorts sometimes show no or even inverse correlation (Falk 2010; Steiger 2011; Töglhofer et al. 2011). In some cases, there are also interactions between neighboring areas, with higher-elevation ski areas in particular experiencing an influx of skiers during winters with little snow, as winter sports activities are not possible at lower elevations (Gonseth 2013; Koenig and Abegg 1997; Mayer et al. 2018). Another influencing factor in this relationship is the timing of various phenomena: for instance, high snow levels in early winter have a positive effect on ski tourism (Falk and Hagsten 2016; Falk and Vieru 2017), and there is a positive correlation if the Easter vacation take place relatively early in the year (Falk 2010). Other studies have focused on less considered aspects within this topic: according to Malasevska et al. (2017), the relationship between the daily number of visitors and the wind chill temperature in ski resorts in Norway is not linear, and following Hamilton et al. (2007) urban weather also has an influence on ski tourism. Finally, it should be noted that increasingly advanced artificial snowmaking in ski resorts significantly minimizes the relationship of natural snow conditions and skier numbers (Falk and Lin 2018; Gonseth 2013).

One of the main problems in this area of research is that often only monthly or seasonal winter and ski tourism numbers are evaluated and the dynamics of daily changing conditions are not captured because daily numbers do not (publicly) exist or are not recorded (except Mayer et al. 2018). One way to address this issue is to evaluate freely available webcam data (see also Staab et al. 2021) on a daily basis. This has already been applied in other tourism sectors, as pedestrian detection in downtown areas (Szczepanek 2020) and analysis of beach tourism (Gómez-Martín and Martínez-Ibarra 2012; Guillén et al. 2008; Ibarra 2011; Kammler and Schernewski 2004; Moreno et al. 2008; Rodríguez-Toubes et al. 2020; Zhang and Wang 2013). The advantage of this detection method over conventional frequency data (such as sold ski/parking tickets) is on the one hand the more precise spatial and temporal recording of people: webcam data can analyze specific areas of interest, while temporal patterns in frequentation can also be evaluated if the temporal resolution is high enough. On the other hand, this methodology can also generate frequentation data for places that are not connected to any (payment) infrastructure (e.g., non-paying parking lots and hiking trails), and thus, no other data is available. Another striking aspect is that daily tourist figures for winter tourism analyze exclusively skiers and snowboarders in this context. This is an important gap, especially given the supposedly rising importance of non-skiing activities, or to be more precise, activities beyond paying skiing guests (e.g., ski touring on ski slopes or hiking). Combining these two research gaps, this approach can provide added value to various research issues due to the new perspective, e.g., assessing the effects of climate change on winter/ski tourism demand patterns or sustainability monitoring at relevant touristic destinations.

In this study, we use for the first time (to our knowledge) the approach to derive daily visitor numbers — which contain a clear added value compared to monthly/annual numbers in understanding tourist behavior — of different tourist types of winter tourism by means of webcam images. In a second step, we analyze the association with meteorological and time-related variables. Within the study, the aim is to answer the following research questions:

1. To what extent do time-related and meteorological variables influence daily visitor numbers in winter, and can these relationships describe the character of a tourist destination?
2. What influence do time-related and meteorological variables in combination have on daily visitor numbers in winter and, if applicable, can more and less important factors for the number of visitors be determined?

## Materials and methods

### Study area and period

**Fig. 1:**
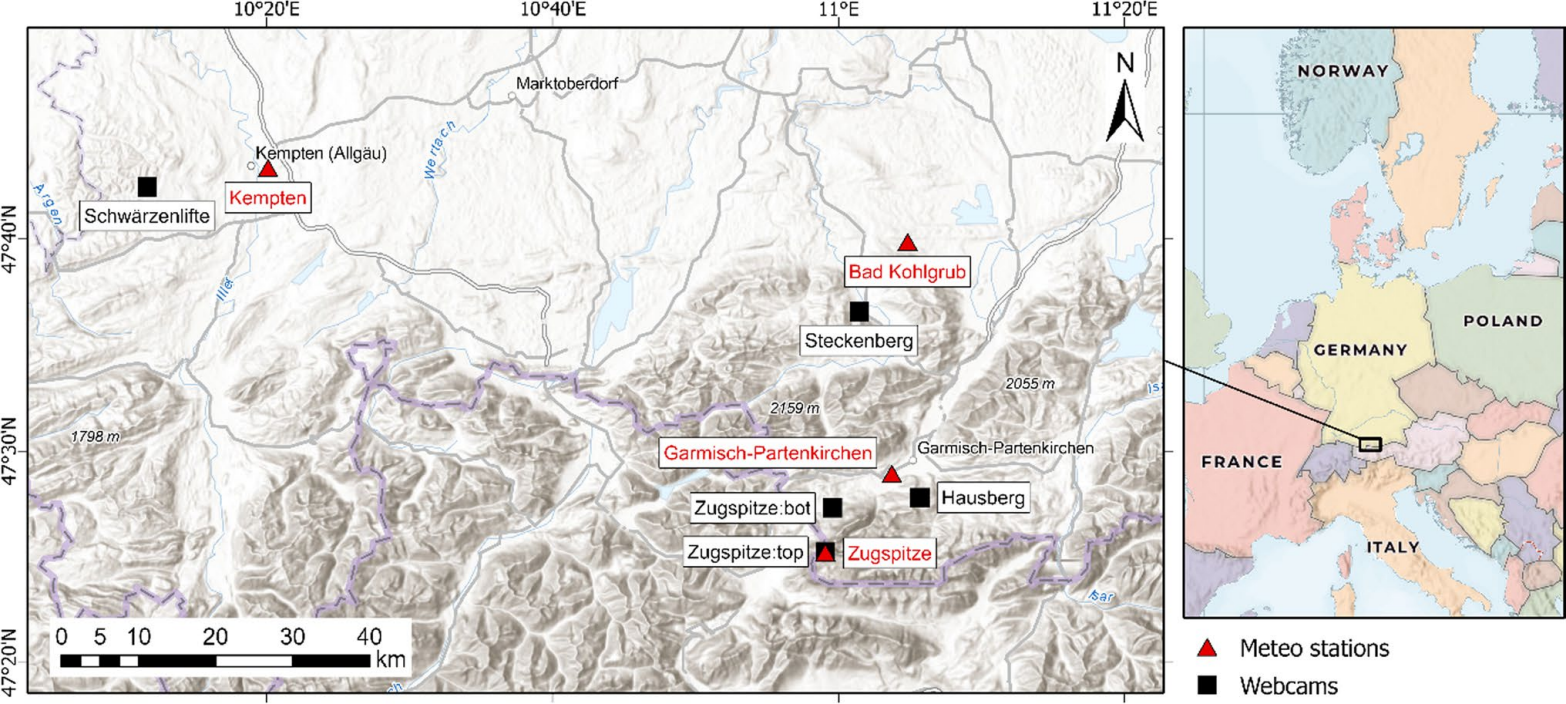
Overview map of the five analyzed webcams and four nearby meteorological stations (left) along with their location in Europe (right; data source: Esri, FAO, NOAA, USGS; HERE, Garmin, Foursquare, METI/NASA)

A total of five webcams were selected for this study, with all cameras installed in the Bavarian Alpine region. The webcam sites were selected by the criteria of (mostly) gapless data availability from 2018 to 2022 and appropriate image detail for person/car detection. Within the Bavarian Alps, tourism plays an important role in the regional economy, with a contribution to national income in the Alpine counties of at least 5% and in some cases more than 15% (Soboll et al. 2012). The webcams of Zugspitze (summit and valley), Hausberg, and Steckenberg are located in the Zugspitz Region (Fig. 1). The Zugspitz Region is one of the most popular tourist destinations in Bavaria. Per year, the region counts over 5 million overnight stays (Zugspitz Region GmbH 2023). The ski resort Zugspitze is the only glacier ski resort in Germany with 20 km of slopes. The lifts are operated from December until May (Bayerische Zugspitzbahn Bergbahn AG 2023b). The “Zugspitze:top” (Radlherr & Keuschnig 2023d) camera is located at the summit observation deck, whereas the second camera (“Zugspitze:bot”; Radlherr and Keuschnig 2023c) is at the (bottom) valley station of the Eibseebahn. This camera is the only one that is not analyzed by the number of people, but by the number of vehicles, as it records the parking lot of the Eibseebahn. In addition to the Zugspitz ski area, the Garmisch-Classic ski area, which offers 40 km of slopes and is operated from December to April, is in close proximity (Bayerische Zugspitzbahn Bergbahn AG 2023a). Within this ski area, the third camera (“Hausberg”; Radlherr and Keuschnig 2023a) is located at the Hausbergbahn top station.

The fourth webcam (“Steckenberg”; Panomax GmbH 2023) of the Zugspitz region is mounted in the ski resort Steckenberg. The ski area is located near Unterammergau and, with 5 km of slopes; it is one of the smaller ski areas in the region (Steckenberg Erlebnisberg Unterammergau 2023). The fifth webcam (“Schwärzenlifte”; Radlherr and Keuschnig 2023b) provides images of the Schwärzenlifte Eschach ski area, with 3 km of slopes (bergfex GmbH 2023). This ski area is located in the Allgäu region, about 10 km west off Kempten. The webcam results were merged with weather information of the German Meteorological Service (Deutscher Wetterdienst, DWD). To ensure the most accurate representation of the weather conditions at the camera locations, the climate stations with the shortest distance to the webcams were chosen. A detailed list is given in Table 1.

**Table 1:** Location and altitude of the five analyzed webcams and four climate stations and their corresponding distances. Climate stations are described by altitude (alt), latitude (lat), and longitude (long; Deutscher Wetterdienst 2022)

Webcam	Location	Altitude (in m)	Climate station (location, alt, lat, long)	Distance to webcam (in km)
Zugspitze:top	Climate station on top of the Zugspitze	2962	Zugspitze, 2964.50 m, 47.4210, 10.9848	0
Zugspitze:bot	Valley station of the Eibseebahn at Eibsee	1000	Garmisch-Partenkirchen, 719.25 m, 47.4830, 11.0621	6
Hausberg	Hausberg top station in the ski resort Garmisch-Classic	1320	Garmisch-Partenkirchen, 719.25 m, 47.4830, 11.0621	3
Schwärzenlifte	Eschach in Oberallgäu	1068	Kempten, 705.20 m, 47.7233, 10.3348	10
Steckenberg	Valley station Steckenberg lift in Unterammergau	860	Bad Kohlgrub, 742.00 m, 47.6652, 11.0805	7

The study covers three winter periods from 2018 to 2022, each from December 1st to March 31st of the following year. The camera at Schwärzenlifte was only evaluated starting from 2019 on due to a different camera angle in the winter of 2018/2019. In addition, the period from December 2020 until March 2021 was excluded from the analysis, since due to COVID-19 pandemic regulations, the lifts were out of service during this period (Deutsche Presse-Agentur GmbH 2021; Schlemmer and Schnitzer 2023; Steiger et al. 2023). As the webcams are located exclusively at lift stations, this effect would override the influence of weather conditions on visitor numbers, as presumably significantly fewer persons would be detected than under non-pandemic conditions. To exclude double counting, only one image per day was analyzed. For the analysis, the time of 1:00 pm was chosen, as all visitors should have arrived at the locations by then and only a few visitors have already departed. For reasons of comparability, the same time was set for all five webcams.

### Data

The climate stations of the DWD provide a range of meteorological variables (Deutscher Wetterdienst 2022). Fresh snow height was additionally calculated as the difference between the snow height on the previous day and the snow height on measurement day. Snow on previous day was selected as a variable with the hypothesis that increased tourism activity can be expected in the ski areas when there is (good skiable) natural fresh snow. An overview of the weather variables used in the study is given in Table 2. In addition to the meteorological variables, the months (December, January, February, March), weekends (yes/no), and Bavarian school vacations (yes/no) were also included in the analysis (Bavarian State Ministry for Education and Cultural Affairs 2023).

**Table 2:** The meteorological variables included in the analysis with their abbreviation and unit

Meteorological variable	Abbreviation	Unit
Mean wind speed	W_Mean_	m/s
Maximum wind speed	W_Max_	m/s
Cloudiness/coverage	COV	1/8 of cover
Precipitation	P_Sum_	mm
Duration of sunshine	SUN	Hours
Snow height	S_H_	cm
Mean temperature	T_Mean_	°C
Minimum temperature	T_Min_	°C
Maximum temperature	T_Max_	°C
Relative humidity	RH	%
Fresh snow height	S_FR_	cm
Snow on previous day	-	Yes/no

### Methods

#### Object detection and computer vision

In this study, the data analysis software R (version 4.2.1) was used, utilizing the “platypus” package, in combination with the “keras” (image classification with Convolutional Neural Networks) and “tensorflow” (Machine Learning) package for object detection. An existing code provided by Maj (2020) was altered for the study, for analyzing “own” pictures, setting different thresholds to improve the recognition rate, or optimizing the output presentation for a more convenient evaluation. Object detection is done by using the one-step detection algorithm YOLO (You only look once). In this study, the third version, YOLOv3, was used containing an architecture with pre-trained COCO (Common Objects in Context) weights. YOLOv3 uses an image size of 320 × 320 pixels. For this study, the image size was altered according to the size of the webcam pictures which is further described in the following section. As the object detection is done at three scales (8, 16, and 32), the image size however must be dividable by 32 (Szczepanek 2020). There are 80 different object classes, which can be identified by the model. Here, only two (“person” and “car”) were relevant.

#### Preparation of webcam material

As a first step, the images were downloaded from the respective websites (see 2.1; with the help of the operators) and cropped. This was necessary to further increase the recognition rate because people/cars in the background of the picture or at a greater distance were recognized less well or not at all. The section of interest was selected in such a way that individual persons/cars could still be recognized as such. In Table S1, the original sizes and the image sections are listed.

**Fig. 2:**
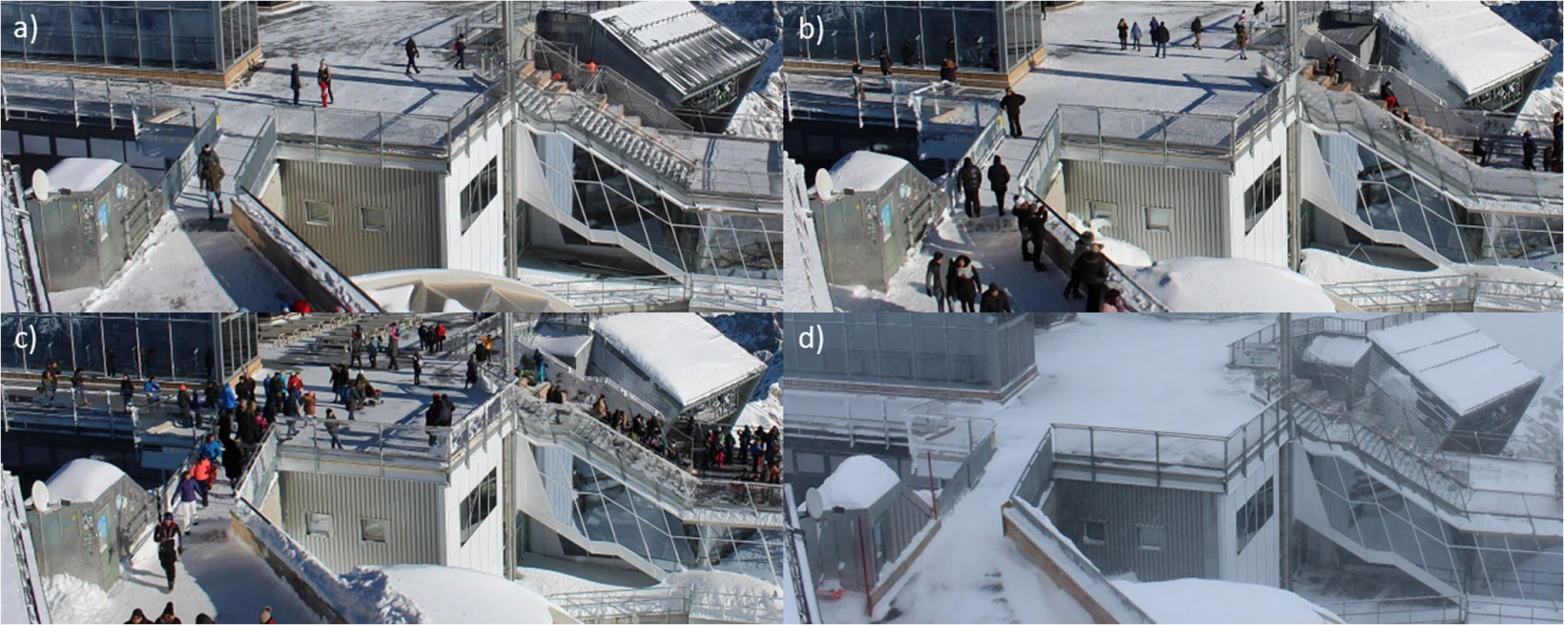
Example pictures of the different density groups for the location “Zugspitze:top” (a: density group “d1”, b: density group “d2”, c: density group “d3”, d: density group “no person”)

As a second step, the webcam cut-outs were sorted and grouped after the density of objects to be analyzed, in order to determine individual model thresholds for the specific density groups and thus obtain better detection results. Gómez-Martín and Martínez-Ibarra (2012) used this technique to sort webcam pictures by people densities at a Spanish beach. In this study, four levels of density were set subjectively and without any specific thresholds: density group “no person/no car”, density group 1 (d1), density group 2 (d2), and density group 3 (d3). The density levels can be defined as follows: (d1) only a few people/cars can be seen, (d2) significantly more people/cars can be seen, but the pictures are not “overcrowded”, (d3) High density of people/cars with many overlaps. In Fig. 2, example pictures of the webcam “Zugspitze:top” are provided. In the case of the webcam Steckenberg for winter 2021/2022, the pictures were additionally grouped by “blurred” and “not blurred”, because a significant proportion of the webcam images were blurred here. In individual cases, pictures had to be assigned to no person/cars because the software did not recognize any persons/cars if the picture quality was too poor or due to unfavorable weather conditions.

After the preparation of the webcam pictures, sample tests for each camera and each density group were conducted. For this purpose, 30 random images of each camera were picked (10 per density group). In the case of Steckenberg, another 24 pictures were pretested because of the blurred density groups in 2021/2022. To be able to compare actual with recognized numbers of the model, the persons/cars on the 30 chosen pictures for each webcam were counted manually, first. In the next step, the numbers were compared with the results of the object detection for the same images. If in more than five cases the deviation was higher than 20% or, in absolute numbers, higher than 5, the recognition thresholds of the YOLOv3 model “obj_threshold” and “nms_threshold” were adapted. The thresholds for each camera are summarized in Table S2, and the final results of the sample tests can be found in Table S3. In this study, in total an amount of 1518 images were analyzed with the detection algorithm. For “Zugspitze:bot”, the highest number of images was available (361), followed by “Zugspitze:top” (356) and “Hausberg” (356). Significantly fewer images were available for the ski areas “Schwärzenlifte” (243) and “Steckenberg” (202). For three locations (“Zugspitze:top”, “Hausberg”, “Steckenberg”), most of the images are (manually) classified as d2. Only at “Zugspitze:bot” (69%, d3) and “Schwärzenlifte” (34%, d1), more images were assigned to another density group. Detailed information about the distribution among the density groups can be found in Table S3.

#### Statistical analysis

In the first step of the analysis, the webcam data were univariately analyzed with both the time-related (boxplots) and the meteorological data (Spearman’s rank correlation). To detect statistically significant differences between subgroups, the Kruskal–Wallis test was applied in the time-related analysis. Furthermore, two-way interactions of all variables within a model analysis were evaluated. In the first step, the appropriate model had to be selected. To model count data two types of models were considered — a Poisson generalized linear model (GLM) or negative binomial (NB) GLM. Since there is a high variance in the data and thus a tendency to overdispersion, an NB was the model of choice (Dunn and Smyth 2018). Since the available data is characterized by many zeros, zero-inflated models were also considered for evaluation. Compared to the zero-inflated Poisson (ZIP) model, the zero-inflated negative binomial (ZINB) model simultaneously considers zero inflation and overdispersion in the numerator of the model. Therefore, this model may be a good choice for this study (Neelon 2019). The Akaike information criterion (AIC) was used as a decision criterion for model selection (Akaike 1998). In addition to the AIC, the significance (*p* value < 0.05) of individual variables was used as a decision criterion.

Before comparing the models, the variables of interest were determined. Of the initially selected meteorological variables (see Table 3), seven were chosen for the model based on the following arguments: temperature and wind variables in the mean, as well as the minimum and maximum in a daily resolution were provided by DWD. However, these were highly correlated and hence only mean wind speed and maximum temperature — as the determining temperature variable of the daily weather forecast — were included. In this study rather low-elevation ski areas (except Zugspitze) were considered, which often must be artificially snowed to maintain operations. Thus, artificial snow is an important factor when it comes to ski tourism activities in winter (e.g., Steiger et al. 2019). However, artificial snow is not considered in this study. Since the correlations of snow height and fresh snow with visitor numbers were low or not significant for most of the sites either, these variables were left out of the multivariable model. In addition to the weather variables, the indicator variables weekends and vacations were included. Months were not considered, as they provided little information about visitor behavior.

**Table 3:** Variables along with corresponding units for the analyzed models

Model variables	Unit	Count part of the chosen model	Zero-inflated part of the model (in case of ZINB)
Weekend	Yes/no	x	x
Vacation	Yes/no	x	x
Mean wind speed	m/sec	x	
Cloudiness/Coverage	1/8	x	
Precipitation	Yes/no	x	x
Duration of sunshine	Hour	x	
Maximum temperature	°C	x	
Relative humidity	%	x	
Snow on previous day	Yes/no	x	x

As a next step, a NB model and a ZINB were set up using all variables listed in Table 3 and for all five locations separately. After model fitting, the AIC of both models was compared. The respectively best models were refined. To obtain a model consisting of only significant variables for each site, the variables with the highest p-value were removed sequentially. For the ZINB models, this procedure was conducted for the zero-inflated part at first, then for the count part. Finally, the AIC of the final models was compared with Poisson and ZIP models for validation and ZINB models were compared with a NB model.

## Results

### Time-related variables

**Fig. 3:**
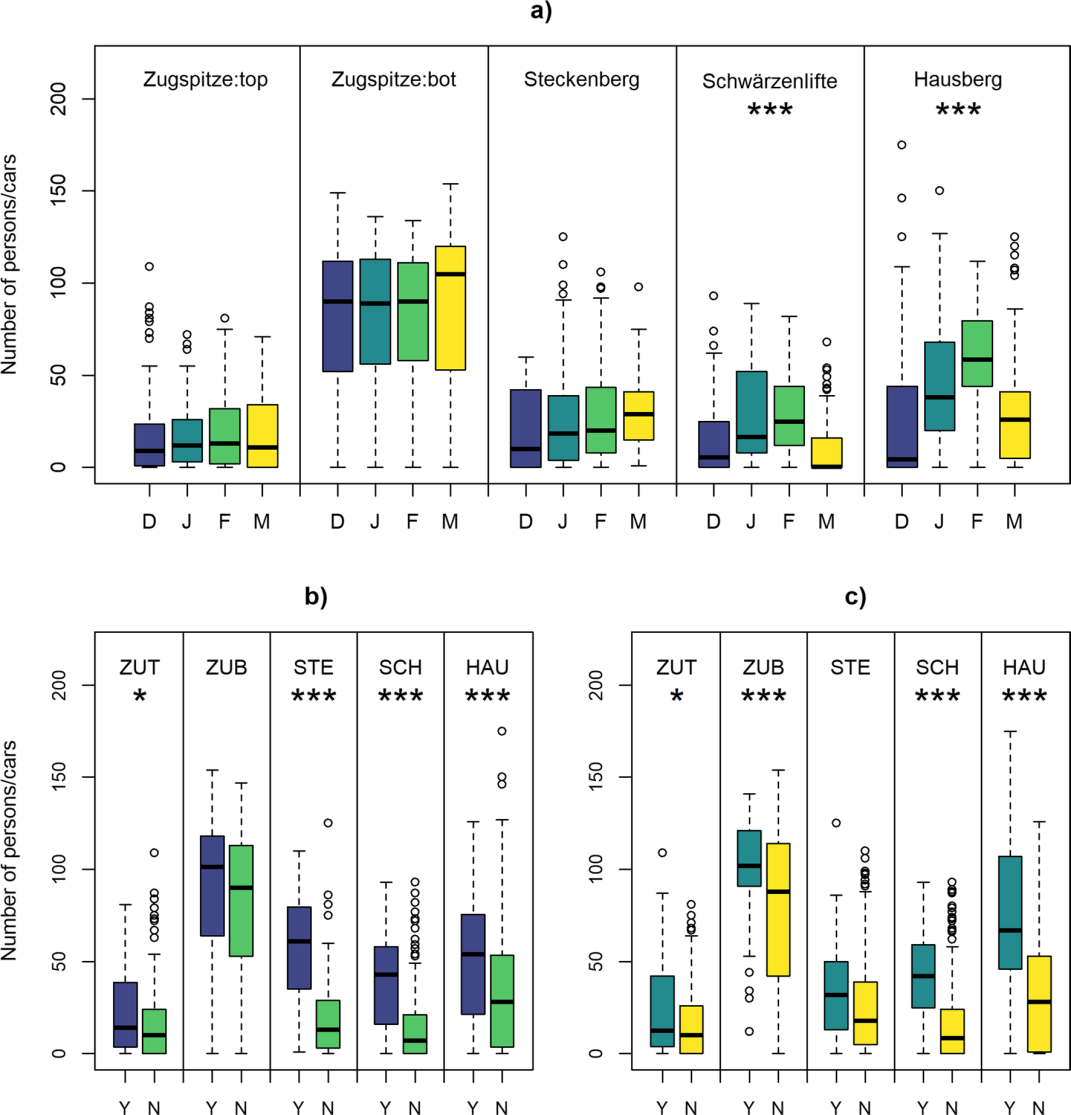
Number of people/cars detected in webcam images classified by **a** months (December, January, February, March), **b** weekends (yes/no), and **c** vacation periods (yes/no). The abbreviated sites in **b** and **c** follow the same ordering as in **a**. Statistically significant differences according to the Kruskal–Wallis test between the respective groups are marked with an asterisk (*p < 0.05; **p < 0.01; ***p < 0.001)

Weather and time-related variables had different effects on visitor numbers at the five sites. In terms to month, the number of people/cars recorded at the Zugspitze was very evenly distributed both on the summit and in the valley (except for March; Fig. 3a). While the ski area at Steckenberg recorded increasing numbers of people per month throughout the winter, the months February and January stood out clearly in terms of numbers at Schwärzenlifte and at Hausberg (4.5 or 13 times more people in February than in December; differences between the individual months were statistically significant: *p* value < 0.001). While the areas observed on top of and at the valley station Zugspitze revealed only minor differences in visitor/car numbers between weekend/no weekend and vacation/no vacation (Fig. 3b and c; except Zugspitze:bot for vacation: *p* value < 0.001), the other ski areas showed greater differences. At Steckenberg (median on weekends: 61; median from Monday to Friday: 13) and at Schwärzenlifte (median on weekends: 43; median from Monday to Friday: 7), more skiers could be seen at the weekend than on a weekday, and also the median at Hausberg was higher at the weekend (54 to 28; at all three sites the differences between the subgroups were statistically significant: *p* value < 0.001). During vacation periods, the number of skiers detected at Steckenberg was slightly higher than during non-vacation periods (median in vacation season: 32; median not in vacation season: 18), whereas at Schwärzenlifte (42 to 8.5) and Hausberg (67 to 28) there is a clear preponderance during vacation periods (differences between the subgroups were statistically significant: *p* value < 0.001).

### Meteorology

**Fig. 4:**
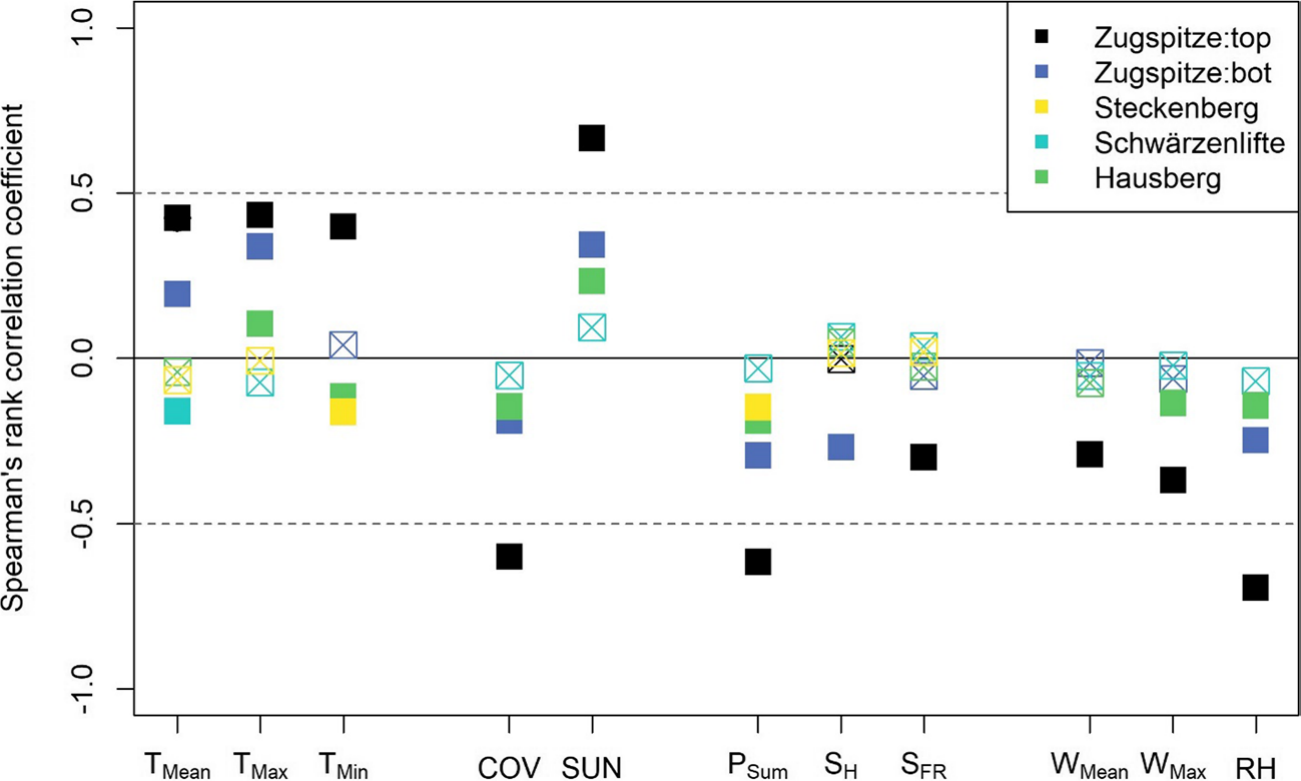
Spearman’s rank correlation between the people/cars detected in the webcam images and the individual daily meteorological variables (*T*_Mean_ = mean temperature; *T*_Max_ = maximum temperature; *T*_Min_ = minimum temperature; COV = cloudiness/coverage; SUN = duration of sunshine; *P*_Sum_ = precipitation; S_H_ = snow height; S_FR_ = fresh snow height; W_Mean_ = mean wind speed; W_Max_ = maximum wind speed; RH = relative humidity). Filled squares indicate a statistically significant correlation (*p* value < 0.05). The exact values can be found in Table S4

Differences were observed in the relationship between the detected persons/cars and the meteorological variables depending on the area (Fig. 4 and Table S4): While statistically significant positive correlations between people/cars and mean/maximum/minimum temperature and sunshine duration could be seen at the Zugspitze summit and at the bottom (Spearman’s rank correlation coefficient: + 0.04 to + 0.43), only correlations between − 0.16 and + 0.10 were retrieved for the other three webcams. Mostly negative correlation coefficients were determined for cloud cover (− 0.60 to − 0.05), precipitation sum (− 0.62 to − 0.03), fresh snow height (− 0.30 to 0.04), mean (− 0.29 to − 0.02), and maximum (− 0.37 to − 0.02) wind speed, as well as relative humidity (− 0.69 to − 0.07). Those correlations were statistically significant mainly for the Zugspitze webcams and Hausberg (exception: *T*_Mean_ and *T*_Min_ at Schwärzenlifte and *T*_Min_ and *P*_Sum_ at Steckenberg). The snow depth correlated only significantly (negative) with the number of detected cars at the Zugspitz valley station (− 0.27).

### Multivariate modeling

**Fig. 5:**
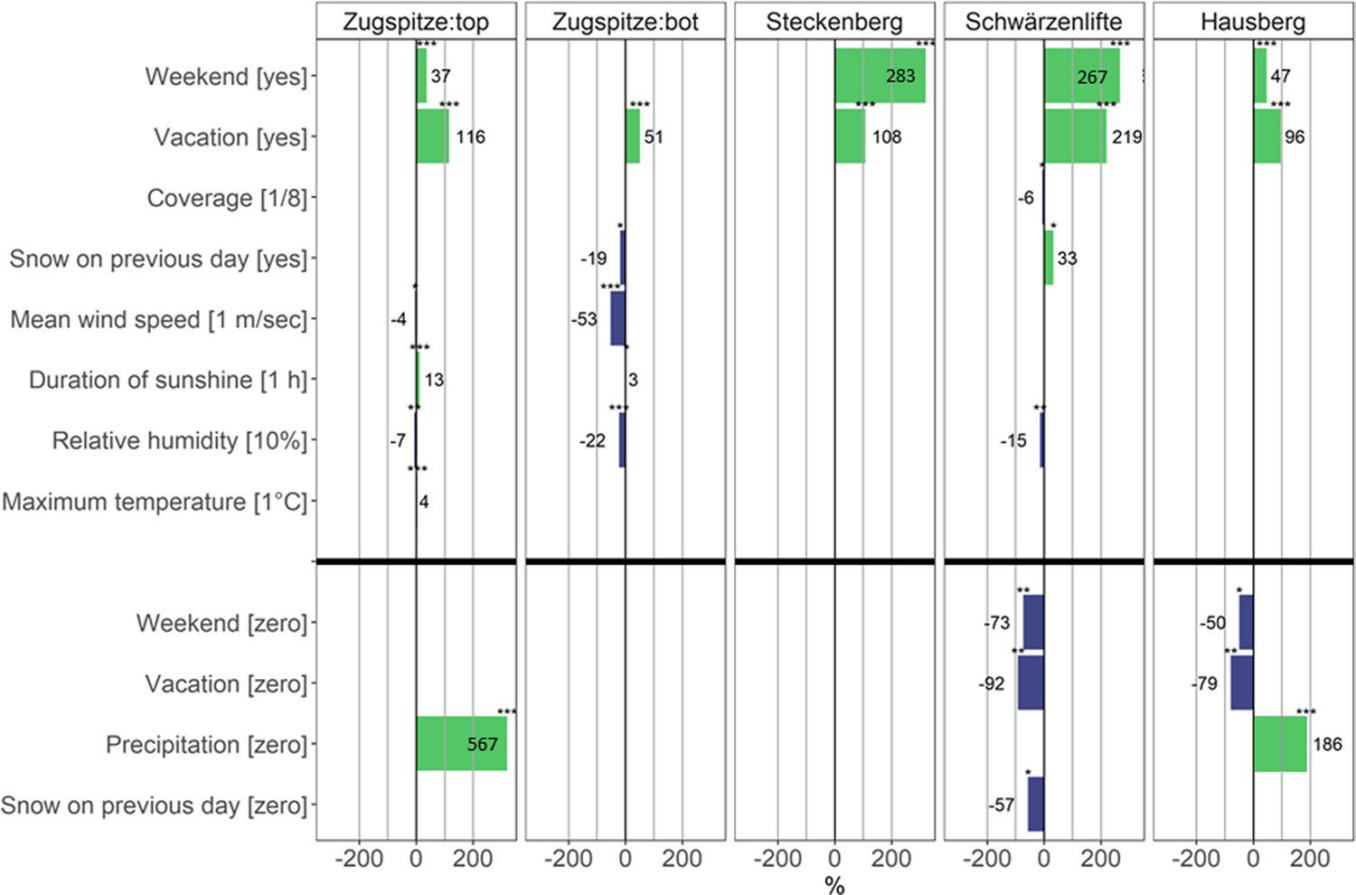
Change of numbers of persons or cars influenced by different meteorological and time-related variables in % (above) and zero-person probability in % (below). The upper part of the figure shows the significant variables of the count parts of the applied models. The lower part, separated by the black bolt line, shows the significant variables of the zero-inflated part of the ZINB. The asterisks describe the significance level of the variables: *p value < 0.05; **p value < 0.01; ***p value < 0.001. For “Steckenberg” and “Zugspitze:bot”, a negative binomial model was applied. Thus, zero inflation was not measured. The respective numerical values can be found in Table S5

Figure 5 summarizes the results of the multivariable analysis. For all webcams studied, the number of people/cars observed increased at weekends (+ 37 to + 283%) and during vacations (+ 51 to + 219%; the only exception is the Zugspitze valley station on weekends). Cloud cover (once − 6% per 1/8 coverage increase) and maximum temperature (once + 4% per °C increase) played only a minor role for most camera sites, while sunshine duration played a relevant role just at the two Zugspitze sites (+ 3/13% per hour increase). Wind speed (− 4 to − 53% per m/sec increase) as well as relative humidity (− 7 to − 22% per 10% increase) had a negative effect in some cases, and snowfall on the previous day had both positive (Schwärzenlifte: + 33%) and negative (Zugspitze:bot: − 19%) effects.

In the zero-inflated part of the ZINB, precipitation had a strong effect (+ 186 to + 567%) on the detection of zero persons in a webcam image in two out of three sites. On the other hand, the factors weekend (− 50 to − 73%) and vacation (− 79 to − 92%) had the opposite effect for Schwärzenlifte and Hausberg and snowfall on the previous day for Schwärzenlifte (− 57%).

## Discussion

Visitor numbers at the five studied sites in the Bavarian Alpine region in winter revealed both similar and divergent patterns with respect to time and weather, depending on the destination. At all sites, more people were present at weekends and during vacations on average. However, mean visitor numbers from winter 2018 to 2022 differed considerably among the locations. Single meteorological variables had a significant impact on the number of visitors only for the two Zugspitze webcams analyzed. Combining all meteorological and non-meteorological factors, it became clear that overall, the time-related variables had a similar or even higher influence on the daily visitor numbers than weather variables. However, each site revealed some unique properties related to its destination.

The monthly distribution of detected persons at the two Zugspitze webcam locations was more uniform than at the other three sites. The summit platform of the Zugspitze is a year-round tourism destination, which suggests a constant flow of visitors throughout the winter. The valley station of the Zugspitze cable car equally mirrored this pattern, except for the observed rise of visitor numbers in March, probably due to warmer weather — and therefore more pleasant weather conditions on the summit platform — and additional arrivals from hikers intending to walk at the Eibsee region. In contrast, the studied “classical” ski resorts had a much more seasonal pattern of detected people in the observed period, with maximum numbers in the main ski months of January and February. In both December and March, due to the absence of snow and temperatures being too high for (economically reasonable) artificial snowmaking, lift operations within the ski areas are partially suspended and thus significantly fewer people visited the ski lift areas. Additionally, another reason for the drop in numbers in March could be the decline in demand after the vacations, which normally take place in late February/early March in Bavaria.

At weekends and during vacation periods, more people were detected at all study sites, which seems logical from the point of view of conventional weekly working days (Monday to Friday) and family vacation schedules (depending on school vacation). However, these patterns here were also weaker at the Zugspitze sites than at the rest of the ski resorts. We assume that, in addition to regional and national day tourists, mainly international tourists from abroad visit the main attraction of the highest mountain in Germany, the Zugspitze, and thus act more independently of the day of the week and Bavarian vacations (Bavarian State Office for Statistics 2023). Other studies within this topic complex confirm winter skiing activities as part of vacation activities: Falk (2010) described that overnight stays at Austrian ski resorts increased by 1.9% on average when Easter vacations occur early, and Falk and Vieru (2017) observed a 9 to 10% increase in ski lift ticket revenue in Finland when Easter vacations occur early in March. Additionally, Malasevska et al. (2017) showed a strong inter-weekly variance in ski area visitors related to weekends in Norway, and Hamilton et al. (2007) a substantial proportion of ski area visitors in the USA operating on weekends or during vacation periods. Finally, Neumair et al. (2022) noted in their analysis of fatal outdoor accidents in the Austrian mountains in winter that weekends significantly increased the odds of these events.

A dichotomy between the two webcams at Zugspitze and the three ones in other ski resorts was also found in the correlation between meteorology and people/cars detected. For almost all analyzed variables, the correlations at Zugspitze were significant: more people are present at higher temperatures, less cloud cover or a lot of sunshine, less precipitation, less fresh snow, less wind, and less humidity, summarized as “nice” weather. This makes sense, since a quite expensive visit to the Zugspitze platform is especially interesting when there is a good distant panoramic view on the neighboring and the central alpine mountains, mostly associated with sunny, warm weather prevailing (see also Mayer et al. 2018). The smaller, but still similar correlations between weather and visitor numbers retrieved at the Eibseebahn valley station can be explained by the fact that visitors (in cars) detected there, may also park there for a valley hike (especially around the Eibsee) and may not consider visiting the summit on less “nice” weather conditions.

At the three locations of “pure” ski resorts (Hausberg, Schwärzenlifte, Steckenberg) studied, on the other hand, visitor numbers were only weakly and mostly insignificantly correlated with meteorological variables. This is interesting from two points of view: first, it seems that people ski regardless of the prevailing weather conditions, likely because most of them want to profit from each day of their pre-booked vacations (especially at Hausberg); second, the current natural snow depth does not play a role either. However, this can be explained by the fact that the measuring stations analyzed only natural snowfall or snow depth, and all the ski resorts considered have artificial snowmaking facilities, which (if the ambient temperature permits) make skiing possible regardless of the current snow conditions.

Small correlations between meteorology and ski tourist numbers were equally reported by other studies (Falk 2013, 2015; Mayer et al. 2018). Bausch et al. (2021) additionally found in a case study in the Bavarian Alps that in winter short-term weather forecasts have no influence on guest arrivals. On the other hand, there are also studies that determine a higher influence of weather conditions on ski tourism. However, some of these mainly analyzed extreme winters with comparatively low (natural) snow depths, significant precipitation deficits and higher temperatures (Koenig and Abegg 1997; Pickering 2011). Comparable extreme weather conditions did not play a dominant role in the 2018 to 2022 study period, which meant that snowmaking and skiing was always possible throughout the season. Studies which, as in this study, analyzed daily visitor numbers, but reported that aspects of meteorology explained ski tourism figures, all used a different methodology (Demiroglu et al. 2015; Gonseth 2013; Hamilton et al. 2007; Malasevska et al. 2017; Shih et al. 2009): usually concrete skier numbers or ski passes sold are analyzed, whereas in this study only persons captured by a webcam at a certain time of day (1 pm), which rather allowed a categorical estimate of the visitors present. Daily recorded skiers or sold ski passes, on the other hand, may provide more precise (financial) numbers; however, a more precise recording of the meteorological effects on real ski tourism activities and frequentation in the landscape (for example, on a certain area, a ski slope) may be related to real camera data.

If both groups of variables (meteorological and non-meteorological) are finally combined in one model, the time-related factors of weekends and vacation periods have a similar or even higher impact on the detected persons/cars than meteorology. Especially for the ski resorts studied, many people were mainly present on Saturday or Sunday or during school vacations (see also Witting and Schmude 2019), whereas meteorology does not play a significant role. This finding is equally supported by our further analysis. In addition, an opposite effect at two locations is interesting here: while there is on average less tourism activity at the valley station of the Zugspitze when it has snowed the day before, the opposite is the case at the Schwärzenlifte. This could be explained by the fact that people maybe frequent lower altitude areas here to profit from the more and more rare fresh natural snow on these days. For the sites that were additionally analyzed with a zero-inflated model, the presence of precipitation at the summit platform of the Zugspitze platform and in the Hausberg ski area is also a decisive factor in whether no people are detected at all (although at Zugspitze you can access the summit, but not enter the platform). This makes sense since both the views on the Zugspitze and the skiing fun at Hausberg are significantly limited in the presence of precipitation and cable car tickets might be too expensive at the end. This is not the case at Schwärzenlifte, which speaks for the previously discussed meteorological independence of skiing at this location. Very few studies have combined daily winter tourist numbers, weekend and vacation periods, and meteorological variables in a multivariable model: However, these few existing studies equally show that weekend and school vacation periods have a very high impact compared to meteorology, especially on ski tourism (Hamilton et al. 2007; Malasevska et al. 2017; Shih et al. 2009).

For operators of winter and especially ski tourism destinations, these results can be used to conclude that ski tourism will be practiced in the future despite changing temperature or precipitation conditions due to climate change, as long as sufficient artificial snow can be made. When renewing tourist infrastructure in ski resorts, it may also make sense to focus more on the “suitability for bad weather”, since a similar frequency of use exists here. From the point of view of the visitor to winter tourism destinations, it makes sense to choose a day during the week or outside of the vacation periods for the visit if low frequency is desired.

Finally, we have to discuss possible limitations and uncertainties within our study. In general, the question arises whether an analysis of three (or two) winters is representative in this context and how the correlations would turn out in a climatically more extreme winter with little snow or higher temperatures. Moreover, when extracting the daily number of visitors via webcam images, only one point in time per day is analyzed, and there is of course a certain degree of randomness in how many people are currently visible on the webcam. In addition, the webcams considered individually were not checked whether 1:00 pm is a representative time for recording the visitors present. Nevertheless, it can be assumed that even one (reasonable) time during the day is sufficient to capture tourism activity (roughly) at different weather and time-related conditions, although evaluating images at different times during a day would likely provide more robust results. Furthermore, the detection methodology itself also contains uncertainties, even if this is already counteracted by optimizing the threshold values in a pre-selection of the images. Also, the image quality of the webcam data is an uncertainty factor, especially in the case of distant people or people in the background of the image. Finally, it must also be mentioned that the type of tourism practiced, or the intention of the individual visitors cannot be recognized or can only be recognized with difficulty on the webcam images (especially at the two study sites at the Zugspitze). About the behavior or the reaction of the visitors to various influencing factors — in contrast to (financially and time-consuming) surveys on site — only assumptions can be made with the help of this methodology.

Concerning meteorology, it is important to note that there are sometimes larger differences in altitude between the webcam locations and the nearby climate stations of the German Meteorological Service. Especially the snow depth, the temperature and the wind speed are altitude-sensitive variables, which can result in considerable differences between the measured and the real values at the webcam, whereby no correction factors were applied. Additionally — as already mentioned in some parts of this study — the factor artificial snow is not reflected in the meteorological data, which can lead to a significant distortion of the measured relationship between the detected number of persons and the snow depth.

## Conclusion

The time-related variables weekend and school vacation influence the number of people or cars present at different Bavarian winter (and ski) tourism sites clearly and in comparison, also partly more than different meteorological variables. Meteorology is relevant in terms of daily visitor numbers mainly at locations that include not only ski tourism but also other forms of winter tourism. In the considered ski areas, on the other hand, skiing is done regardless of the current weather, with the existence of artificial snow playing a major role here. Including the respective month in the analysis, it is possible to develop a characteristic for each winter tourism location based on the data. Thus, in our study, we were able to show that the approach of deriving visitor frequency via automatic person recognition in webcam images, which was applied for the first time in winter tourism (to our knowledge), can be a useful addition to better understand visitor flows and their influencing factors in winter tourism destinations. Furthermore, it was shown that each study site should be considered individually, since even within one destination (e.g., Zugspitze) different results can emerge from the same analysis. Finally, particularly at sites in winter, where no frequentation data are available (destinations and attraction points characterized by mainly non-skiing activities), this methodology can be of great added value for visitor monitoring in outdoor recreation areas and provide a better information base for policy makers as well as employees in tourism and nature conservation.

### Supplementary information

Below is the link to the electronic supplementary material.

Supplementary file1 (DOCX 45 KB)

## Data Availability

The datasets generated during and/or analyzed during the current study are available online at https://www.foto-webcam.eu/webcam/hausberg/ (webcam “Hausberg”), https://www.foto-webcam.eu/webcam/schwaerzenlifte/ (webcam “Schwärzenlifte”), https://www.foto-webcam.eu/webcam/bzb-tal/ (webcam “Zugspitze:bot”), https://www.foto-webcam.eu/webcam/zugspitze/ (webcam “Zugspitze:top”), https://oberammergau.panomax.com/steckenberg (webcam “Steckenberg”) and https://opendata.dwd.de/climate_environment/CDC/observations_germany/climate/daily/kl/ (meteorological data).
