# Supplementary material for: Weather, weekday, and vacation effects on webcam recorded daily visitor numbers in the alpine winter season
Source: Int J Biometeorol. 2023 Dec 1;68(2):305–16. doi: 10.1007/s00484-023-02591-4 (PMC10794479; doi:10.1007/s00484-023-02591-4)
Supplement: Supplementary file 1 — Supplementary file1 (DOCX 45 KB) [file 484_2023_2591_MOESM1_ESM.docx]

**Weather, weekday, and vacation effects on webcam recorded daily visitor numbers in the alpine winter season**

Simon Kloos^1,*^, Carina Bigalke^1^, Matthias Neumair^2^, Annette Menzel^1,3^

^1^TUM School of Life Sciences, Ecoclimatology, Technical University of Munich; Freising, 85354, Germany; simon.kloos@tum.de

^2^TUM School of Life Sciences, Life Science Systems, Technical University of Munich; Freising, 85354, Germany

^3^Institute for Advanced Study, Technical University of Munich; Garching, 85748, Germany; annette.menzel@tum.de

^*^Correspondence: simon.kloos@tum.de; ORCID iD: 0000-0002-9242-1456

Table S1: Description of the image sizes used for object detection. For Steckenberg, the image size changed from December 2021 on.

| Webcam | Original image size | Cut-out image size + distance from left + distance from top |
| --- | --- | --- |
| Zugspitze:top | 1920x1080 | 1360x680+0+420 |
| Zugspitze:bot | 1920x1080 | 1920x200+0+880 |
| Hausberg | 1920x1080 | 700x300+650+780 |
| Schwärzenlifte | 1920x1080 | 1600x440+300+640 |
| Steckenberg | 6000x2500  2021/2022: 6000x4000 | 4272x1256+1728+1800  2021/2022: 4272x1256+1728+2200 |

Table S2: Overview of the “obj_threshold” and “nms_threshold” for each webcam. In the case of webcam Steckenberg, the values in the brackets describe the thresholds for the “blurred” density groups.

| Webcam | Thresholds d1 | Thresholds d2 | Thresholds d3 |
| --- | --- | --- | --- |
| Zugspitze:top | obj_threshold: 0.1  nms_treshold: 0.6 | obj_threshold: 0.12  nms_treshold: 0.5 | obj_threshold: 0.1  nms_treshold: 0.5 |
| Zugspitze:bot | obj_threshold: 0.7  nms_treshold: 0.8 | obj_threshold: 0.6  nms_treshold: 0.83 | obj_threshold: 0.5  nms_treshold: 0.82 |
| Hausberg | obj_threshold: 0.2  nms_treshold: 0.66 | obj_threshold: 0.2  nms_treshold: 0.61 | obj_threshold: 0.15  nms_treshold: 0.63 |
| Schwärzenlifte | obj_threshold: 0.4  nms_treshold: 0.76 | obj_threshold: 0.5  nms_treshold: 0.8 | obj_threshold: 0.45  nms_treshold:0.8 |
| Steckenberg | obj_threshold: 0.4 (0.1)  nms_treshold: 0.7 (0.5) | obj_threshold: 0.45  (0.09)  nms_treshold: 0.7  (0.6) | obj_threshold: 0.42 (0.05)  nms_treshold: 0.72 (0.65) |

Table S3: Overview of the number of webcam pictures (overall values including density group 1/2/3/”no person/car” and per density group 1/2/3) and the relative and absolute mean deviation of recognized and counted persons/cars for the applied sample test. In the case of webcam Steckenberg, the values in the brackets describe the values for the “blurred” density groups.

| Webcam | Number of webcam pictures | | Observations per density group and period | | | Mean deviation of recognition | |
| --- | --- | --- | --- | --- | --- | --- | --- |
|  |  |  | 2018/2019 | 2019/2020 | 2021/2022 | relative | absolute |
| Zugspitze:top | 356 | d1 | 42 | 30 | 36 | 41% | 2 |
|  |  | d2 | 45 | 38 | 65 | 9% | 3 |
|  |  | d3 | 6 | 10 | 0 | 5% | 4 |
| Zugspitze:bot | 361 | d1 | 18 | 17 | 4 | 39% | 4 |
|  |  | d2 | 37 | 19 | 11 | 16% | 7 |
|  |  | d3 | 66 | 83 | 101 | 15% | 14 |
| Hausberg | 356 | d1 | 11 | 9 | 21 | 17% | 2 |
|  |  | d2 | 75 | 72 | 64 | 10% | 5 |
|  |  | d3 | 10 | 7 | 15 | 12% | 13 |
| Schwärzenlifte | 243 | d1 | - | 37 | 45 | 27% | 1 |
|  |  | d2 | - | 31 | 46 | 17% | 5 |
|  |  | d3 | - | 13 | 12 | 11% | 8 |
| Steckenberg | 202 | d1 | 31 | 15 | 9 (13) | 9% (39%) | 1 (3) |
|  |  | d2 | 34 | 35 | 9 (11) | 10% (19%) | 4 (7) |
|  |  | d3 | 2 | 0 | 7 (7) | 9% (11%) | 7 (9) |

Table S4: Univariate correlation values (r_s_: Spearman’s rank correlation coefficient; p-value) of the Spearman’s rank correlation analysis between the people/cars detected in the webcam images and the individual meteorological variables (see Figure 4). Statistically significant correlations (p < 0.05) are marked with an asterix.

|  | Zugspitze:top | | Zugspitze:bot | | Steckenberg | | Schwärzenlifte | | Hausberg | |
| --- | --- | --- | --- | --- | --- | --- | --- | --- | --- | --- |
|  | r_s_ | p-value | r_s_ | p-value | r_s_ | p-value | r_s_ | p-value | r_s_ | p-value |
| T_Mean_ | 0.42 | <0.001* | 0.19 | <0.001* | -0.07 | 0.361 | -0.16 | 0.012* | -0.04 | 0.438 |
| T_Max_ | 0.43 | <0.001* | 0.34 | <0.001* | -0.01 | 0.903 | -0.07 | 0.258 | 0.10 | 0.049* |
| T_Min_ | 0.40 | <0.001* | 0.04 | 0.450 | -0.16 | 0.024* | -0.15 | 0.020* | -0.12 | 0.028* |
| COV | -0.60 | <0.001* | -0.19 | <0.001* | - | - | -0.05 | 0.412 | -0.15 | 0.006* |
| SUN | 0.67 | <0.001* | 0.34 | <0.001* | - | - | 0.09 | 0.148 | 0.23 | <0.001* |
| P_Sum_ | -0.62 | <0.001* | -0.29 | <0.001* | -0.15 | 0.041* | -0.03 | 0.623 | -0.19 | <0.001* |
| S_H_ | 0.00 | 0.954 | -0.27 | <0.001* | 0.01 | 0.841 | 0.06 | 0.317 | 0.05 | 0.374 |
| S_FR_ | -0.30 | <0.001* | -0.05 | 0.299 | 0.02 | 0.804 | 0.04 | 0.568 | -0.03 | 0.634 |
| W_Mean_ | -0.29 | <0.001* | -0.02 | 0.767 | - | - | -0.05 | 0.414 | -0.08 | 0.155 |
| W_Max_ | -0.37 | <0.001* | -0.06 | 0.232 | - | - | -0.02 | 0.736 | -0.14 | 0.011* |
| RH | -0.69 | <0.001* | -0.25 | <0.001* | - | - | -0.07 | 0.279 | -0.14 | 0.007* |

Table S5: Numerical values for the models displayed in Figure 5 for the five tourism destinations considered (Est. = Estimates). The lower two lines show the AIC value for the initial model (NB: negative binomial; ZINB: zero-inflated negative binomial) as a decision criterion. Although the AIC for ZINB was lower for “Zugspitze:bot“ and “Steckenberg” compared to NB, the final decision was made in favour of the NB model, since no statistical significant estimates were available in the zero-inflated part of the ZINB models. The asterisks describe the significance level of the variables: * = p-value < 0.05; ** = p-value < 0.01; *** = p-value < 0.001.

|  | Zugspitze:top | | Zugspitze:bot | | Steckenberg | | Schwärzen-lifte | | Hausberg | |
| --- | --- | --- | --- | --- | --- | --- | --- | --- | --- | --- |
|  | Est. | p-value | Est. | p-value | Est. | p-value | Est. | p-value | Est. | p-value |
| Weekend | 0.315 | <0.001  *** | - | - | 1.344 | <0.001 *** | 1.301 | <0.001 *** | 0.387 | <0.001 *** |
| Vacation | 0.768 | <0.001 *** | 0.412 | <0.001 *** | 0.731 | <0.001 *** | 1.159 | <0.001 *** | 0.674 | <0.001 *** |
| COV | - | - | - | - | - | - | -0.065 | 0.012 * | - | - |
| Snow on previous day | - | - | -0.213 | 0.012 * | - | - | 0.282 | 0.025 * | - | - |
| W_Mean_ | -0.037 | 0.019 * | -0.749 | <0.001 *** | - | - | - | - | - | - |
| SUN | 0.119 | <0.001 *** | 0.032 | 0.040 * | - | - | - | - | - | - |
| RH | -0.007 | 0.008 ** | -0.025 | <0.001 *** | - | - | -0.016 | 0.004 ** | - | - |
| T_Max_ | 0.044 | <0.001 *** | - | - | - | - | - | - | - | - |
| Weekend [zero] | - | - | - | - | - | - | -1.298 | 0.003 ** | -0.685 | 0.037 * |
| Vacation [zero] | - | - | - | - | - | - | -2.489 | 0.003 ** | -1.569 | 0.002 ** |
| Precipitat-ion [zero] | 1.898 | <0.001 *** | - | - | - | - | - | - | 1.049 | <0.001 *** |
| Snow on previous day [zero] | - | - | - | - | - | - | -0.851 | 0.024 * | - | - |
| AIC (NB) | 2454.483 | | 3832.720 | | 1626.077 | | 1791.410 | | 3230.048 | |
| AIC (ZINB) | 2363.367 | | 3806.338 | | 1597.975 | | 1721.141 | | 3029.438 | |
